# A Machine Learning Approach to the Differentiation of Functional Magnetic Resonance Imaging Data of Chronic Fatigue Syndrome (CFS) From a Sedentary Control

**DOI:** 10.3389/fncom.2020.00002

**Published:** 2020-01-29

**Authors:** Destie Provenzano, Stuart D. Washington, James N. Baraniuk

**Affiliations:** Baraniuk Lab, Department of Medicine, Georgetown University Medical Center, Washington, DC, United States

**Keywords:** functional magnetic resonance imaging (fMRI), Chronic Fatigue Syndrome (CFS), logistic regression, machine learning, recursive feature elimination (RFE)

## Abstract

Chronic Fatigue Syndrome (CFS) is a debilitating condition estimated to impact at least 1 million individuals in the United States, however there persists controversy about its existence. Machine learning algorithms have become a powerful methodology for evaluating multi-regional areas of fMRI activation that can classify disease phenotype from sedentary control. Uncovering objective biomarkers such as an fMRI pattern is important for lending credibility to diagnosis of CFS. fMRI scans were evaluated for 69 patients (38 CFS and 31 Control) taken before (Day 1) and after (Day 2) a submaximal exercise test while undergoing the n-back memory paradigm. A predictive model was created by grouping fMRI voxels into the Automated Anatomical Labeling (AAL) atlas, splitting the data into a training and testing dataset, and feeding these inputs into a logistic regression to evaluate differences between CFS and control. Model results were cross-validated 10 times to ensure accuracy. Model results were able to differentiate CFS from sedentary controls at a 80% accuracy on Day 1 and 76% accuracy on Day 2 (**Table 3**). Recursive features selection identified 29 ROI's that significantly distinguished CFS from control on Day 1 and 28 ROI's on Day 2 with 10 regions of overlap shared with Day 1 (**Figure 3**). These 10 shared regions included the putamen, inferior frontal gyrus, orbital (F3O), supramarginal gyrus (SMG), temporal pole; superior temporal gyrus (T1P) and caudate ROIs. This study was able to uncover a pattern of activated neurological regions that differentiated CFS from Control. This pattern provides a first step toward developing fMRI as a diagnostic biomarker and suggests this methodology could be emulated for other disorders. We concluded that a logistic regression model performed on fMRI data significantly differentiated CFS from Control.

## Introduction

Chronic Fatigue Syndrome (CFS) is a debilitating condition estimated to affect at least 1 million individuals in the United States that causes $9.1 billion in annual losses in productivity (Centers for Disease Control Prevention, 2006). CFS is characterized by chronic persistent fatigue that is not alleviated with rest as well as pain, cognitive dysfunction, sleep abnormalities, and symptom relapse after minimal exertion (post-exertional malaise) (Fukuda et al., 1994; Carruthers et al., 2003; Centers for Disease Control Prevention, 2006; Committee on the Diagnostic Criteria for Myalgic Encephalomyelitis/Chronic Illness, 2015).

Controversy persists about the underlying etiology and pathophysiology of CFS, and there remains a need for objective measures of dysfunction to distinguish CFS from psychosocial etiologies like neurasthenia and depression (Pichot, 1994; Pearce, 2006; Committee on the Diagnostic Criteria for Myalgic Encephalomyelitis/Chronic Illness, 2015). Functional magnetic resonance imaging (fMRI) of the brain has shown to be a promising diagnostic tool because CFS subjects may have reduced gray matter thickness and cortical volume losses compared to age-matched controls (Okada et al., 2004), utilize more frontal and parietal regions during cognitive tasks than age-matched controls (cognitive compensation) (Lange et al., 2005), and have different activation patterns when making mistakes (De Lange et al., 2004). CFS subjects are less responsive than age-matched controls on tasks of auditory responsiveness (Tanaka et al., 2006) and demonstrate additional dysfunction following a light exercise task that may provide evidence for post-exertional malaise (Cook et al., 2017). Performance on n-back tasks indicated dysfunction on working memory (Caseras et al., 2006). The results of these studies provided a rationale to investigate if fMRI and the post-exertional malaise experienced by subjects with CFS could differentiate CFS subjects from a sedentary control.

Standard fMRI analysis seeks to compare univariate regions of brain activation at rest or during a task between CFS and control groups. However, multivariate classification methods have become an increasingly popular tool for identifying patterns of brain activity that can differentiate disease physiology (Cox and Savoy, 2003; Kriegeskorte et al., 2006; Haynes et al., 2007; De Martino et al., 2008; Ryali et al., 2010). Machine learning algorithms such as logistic regression, support vector machines, and random forests combined with feature selection can be applied to clustered voxel data to determine patterns of brain regions that may characterize a disorder (Mourão-Miranda et al., 2005; Pereira et al., 2009). We hypothesized that an acute physiology stressor such as a light exercise task combined with the implementation of a machine learning algorithm would allow us to identify a pattern of predominant behavior during fMRI scanning of CFS subjects while performing the n-back memory paradigm.

Subjects underwent fMRI scans on consecutive days while performing the continuous version of the n-back working memory test before (Day 1) and after (Day 2) a bicycle exercise stress test (Rayhan et al., 2013). Blood oxygenation level dependent (BOLD) signals were compared between groups on both days. We followed a standard approach of predictive model building for fMRI data involving feature extraction, model build, validation, and evaluation of performance (Sen et al., 2018). Voxel maps of activations from each subject were mapped to the Automatic Anatomical Labeling (AAL) atlas^AAL^ using SPM12^SPM^. Predictive model features were created from the number of significantly activated voxels for each AAL region for each subject run through a recursive features selection algorithm to identify importance. Data points were iteratively split into training and testing sets to create a logistic regression model (training set) and then validate the results (testing set). Model results were cross-validated to ensure performance. The output of this model was a multivariate pattern of activation that signified the cognitive differences between groups of CFS and sedentary control subjects. This strategy differs from the traditional fMRI analysis technique that quantify significant BOLD differences on voxel-by-voxel and regional basis. The outcomes provide a proof of concept for the implementation of a machine learning algorithm on fMRI data to create a diagnostic tool for CFS.

## Methods

### Ethics

Subjects gave written informed consent for participation and use of all data for publication purposes. Studies were approved by the Georgetown University Institutional Review Board (IRB 2009-229, 2013-0943, 2015-0579) and U.S. Army Medical Research and Material Command (USAMRC) Human Research Protection Office (HRPO A-155547.0, A-18749), and registered on clinicaltrials.gov as NCT01291758, NCT03560830, and NCT03567811. All clinical investigations were conducted according to the principles expressed in the Declaration of Helsinki.

### Subjects

Data was collected from candidates who responded online or by phone or personal contact. Telephone screening after verbal informed consent was performed with 216 subjects, but 105 declined to participate or were excluded from participation after protocol explanation and assessment of chronic medical and psychiatric disease (Jones et al., 2009; Nater et al., 2009). Chronic Fatigue Syndrome was assessed by 1994 Fukuda CDC criteria by having 6 months of debilitating fatigue without medical or psychiatric cause plus at least for of the following eight criteria: problems with memory or concentration, sore throat, sore lymph nodes, myalgia, arthralgia, headache, sleep disturbance, and post-exertional malaise (Fukuda et al., 1994). Veterans with Gulf War Illness were examined by the same process and were excluded (Steele, 2000; Haynes et al., 2007).

Subjects were admitted to the Georgetown Howard Universities Clinical Translation Science Clinical Research Unit and were tested for the N-back working memory task in a 3T MRI scanner on two separate days. They underwent their first fMRI scan and N-back working memory task after overnight rest and then performed a submaximal exercise stress test. Subjects cycled at 70% of age-predicted maximum heart rate (220-age) for 25 min, the ramped up their effort to reach 85% of predicted heart rate. On the next day they had that same submaximal exercise test followed by the second fMRI scan with n-back testing. This study reports on 38 subjects with Chronic Fatigue Syndrome and 31 sedentary controls.

### N-Back Task

Subjects practiced the complete n-back task with blocks of 0-back and 2-back loads in a mock scanner until they felt satisfied with their performance. fMRI data were collected on the non-exercise day (Day 1) and about 1 h after the second submaximal bicycle exercise stress test (Day 2).

The continuous version of the verbal N-back task is a challenging test of subject attention, memory, retrieval, and updating (Owen et al., 2005; Rayhan et al., 2013). Each 1 min long block had three components: 0-back task, 2-back task, and fixation between tasks. Subjects began each block with fixation by viewing a blank screen for 8 s. They proceeded to 0-back testing by viewing a string of nine letters (A, B, C, D) presented in random order for 2 s per letter. Subjects used both hands to press the button on a fiber-optic button box (ePrime software) that corresponded to the letter being viewed^1^. After another fixation period, they viewed a second string of nine letters for the 2-back task. Subjects had to remember the 1st and 2nd letters. When the 3rd letter was presented, they had to press the button corresponding to the letter seen “2-back” (the 1st letter seen 4 s before). The task was designed such that subjects orient, reorder, and engage their working memory to focus their attention in preparation for the next letter. Subjects used individual strategies to remember single letters in series (e.g., A-B-C-D) or through “chunks” (AB-BC-CD, or ABC-BCD). The 1-min blocks were repeated five times which produced time-series scans for 45 letters for 0-back stimulus response measurements (five blocks × nine responses) and 35 responses for the 2-back task (five blocks × seven responses each).

### Functional Magnetic Resonance Imaging (fMRI) Data Acquisition

fMRI acquisition was performed in a Siemens 3T Tim Trio scanner equipped with a transmit-receive body coil and a commercial 12-channel head coil array. Structural 3D T1-weighted Magnetization Prepared Rapid Acquisition Gradient Echo (MPRAGE) image parameters were: TR/TE = 1,900/2.52 ms, TI = 900 ms, field-of-view(FoV) = 250 mm, 176 slices, slice resolution = 1.0 mm, and voxel size 1 × 1 × 1 mm. Functional T2^*^-weighted gradient-echo planar imaging (EPI) parameters were: number of slices = 47, TR/TE = 2,000/30 ms, flip angle = 90°, matrix size = 64 × 64, FoV = 205 mm^2^, and voxel size = 3.2 mm^2^ (isotropic).

### Data Pre-processing

BOLD data was pre-processed through the default pipeline of the CONN version 17 toolbox (Whitfield-Gabrieli and Nieto-Castanon, 2012). Data underwent processing and spatial smoothing with a spatially stationary Gaussian filter of 6 mm full-width half maximum (FWHM) size through the SPM12 software (http://www.fil.ion.ucl.ac.uk/spm/software/spm12/). SPM12 was used to account for movement artifacts between scans and functional anatomic differences not otherwise already compensated for. Spatially normalized images were converted into the Montreal Neurological Institute (MNI) standard stereotactic space (Mazziotta et al., 1995). Pre-processing included a slice-timing correction, outlier detection for Framewise Displacement based on Artifact Detection Tools, and realignment and unwarping of functional images. Spatial normalization resulted in a voxel size of 2.0 mm^3^ (isotropic).

Preprocessed EPI data from individuals were modeled with the following events: instruction, fixation, 0-back, and 2-back. The 2-back > 0-back contrast was analyzed by one-sample *t*-test with motion parameters as covariates of no-interest. The residual 2-back > 0-back condition identified voxels that were significantly more activated during the high cognitive load 2-back than the low cognitive load 0-back periods. The optimal threshold *t*-value to identify significantly activated voxels was determined by plotting the number of significant voxels per subject as a function of *T*-values. The *T* value of 3.17 (*p* < 0.001 uncorrected) was selected.

Voxel data from the T-statistic maps were charted to MNI coordinates and grouped into regions defined by the Automated Anatomical Labeling Atlas (AAL) (Tzourio-Mazoyer et al., 2002) using a custom MATLAB program and functions from SPM12 and xiView 9.6^2^. The AAL atlas was chosen due to its widespread use and recognizability in SPM12, python, and the general fMRI community. The catalog of AAL regions with centers of mass and voxels per region was shown in Table S1 and Figure S1^3^. The numbers of significant voxels per AAL region for each individual were the independent input variables that were fed into the feature selection process and logistic regression learner model. Our approach utilized a machine learning algorithm applied to the 3-D matrix of voxel data split using the binary outcome variable of CFS vs. control status.

### Feature Extraction

Model features (AAL regions with total activated voxels) were selected by a multistep feature reduction process.

Pearson's correlation coefficients were used as a preliminary variable selection methodology to determine highly correlation regions of brain activity. The number of significant voxels in every AAL region in the entire dataset (Testing + Training) was compared to every other AAL region to determine multicollinearity or which regions, if any, could be linearly predicted from the others with a substantial degree of accuracy. This created a matrix of correlations depicting Pearson's Correlation Coefficient for every region. When regions have a Pearson's Correlation Coefficient (R) of ≥0.9, it can be assumed that multicollinearity exists and that these regions should be removed or combined. Multicollinearity may not affect the overall predictive power of a model, but can impact the residual calculations of individual predictors and render the overall coefficients invalid (Belsley, 1991; O'Brien, 2007). Perfect multicollinearity causes the design matrix to have one less full rank and does not allow the ordinary least squares estimator to be inverted (Farrar and Glauber, 1967). Eliminating multicollinearity prevents against inaccurate machine learning algorithms, excessive standard errors for coefficients, and overfitting of models (Kumar, 1975; O'Hagan and McCabe, 1975). Multicollinearity was tested for three times on the training set, testing set, and training and testing set combined due to the small number of samples to ensure no multicollinearity existed for any combination of variables and that ordinary least squares (OLS) estimators could be obtained. The matrix depicting the training and testing set is depicted in the results.

Next the list of variables for model inputs was reduced using recursive feature elimination (RFE). Only data from the training set was fed into RFE and later, the logistic regression model. RFE is a feature selection method that fits a model by removing the weakest model input (feature) until a specified number of attributes remains or total accuracy level is reached. By eliminating a small number of inputs per loop in an iterative process, RFE attempts to reduce variable dependencies and collinearity that could otherwise impact a model. This data reduction step used the default recursive feature elimination (RFE) algorithm in the scikit-learn python package^4^. The principle of Occam's razor governs that the simplest set of inputs into a machine learning algorithm often leads to the most accurate result, as such this process attempted to whittle down the variables to as few as possible while still controlling for accuracy (Gauch, 2003).

Recursive feature elimination is a greedy feature elimination algorithm similar to sequential backward selection as found in a stepwise logistic regression. It was ultimately determined to use recursive feature elimination to remove excess inputs rather than use stepwise logistic regression to decrease bias in *R*^2^ values, increase standard errors of the parameter estimates, increase confidence intervals, increase *p*-values, and unbias parameter estimates. Stepwise logistic regression can also exacerbate collinearity problems, which was especially important to account for given the small sample size.

### Predictive Model Build

Multiple predictive models were initially tested and evaluated before final presentation of results. These included a Support Vector Machine (SVM), Random Forest, Decision Tree, and Neural Net. Logistic Regression was the algorithm ultimately selected as it fast to build, repeatedly produced the most accurate and generalizable results, and is easy to implement in practice. Logistic regression is an algorithm used to determine the probability of a binary response to be dependent on one or more independent input variables (Walker and Duncan, 1967). A logistic regression works by attempting to fit a model that minimizes coefficients assigned to model inputs and maximizes total differentiated subgroups that fall into the classification region. Coefficients estimate the logarithm of the odds (log-odds) for a dependent variable based on the independent variables (Biondo et al., 2000). Corresponding coefficients for input variables are “regressed” from the data (Freedman, 2009). The model fits the data to the logit equation:

$$\begin{array}{l} \text{p}\left(\text{x}\right)=1/(1+{\text{e}}^{-\left(\beta 0+\beta 1x1+\beta 2x2\ldots +\beta ixi\right)}) \end{array}$$

where β_0_ is the intercept (constant term), and β_1_ and β_2_ are the coefficients for variables x_1_ and x_2_, and β_i_ represents coefficients for all subsequent variables (http://www.alivelearn.net/xjview/). Features fed into the model are assigned a coefficient that is reduced according to stochastic gradient descent until the best possible model (highest accuracy) remains. Stochastic gradient descent is a first order optimization algorithm that seeks to find the minimum of a function by taking steps proportional to the negative gradient of the function at every point (Barzilai and Borwein, 1998). The model was trained on a subgroup of the total dataset that was split into a stratified sample of disease and control subjects and tested on the remaining subgroup. This created a designated “training” and “testing” set. Each testing set was a distinct validation set created for each training set that did not overlap with the training set used to build the predictive model. Recursive feature elimination was run before each predictive model build on each respective training set. The ratio of training to test subjects was varied from 50:50 to 90:10 with the grouping of 70:30 selected to give the optimal model validation. This optimal ratio was determined by evaluating the final predictive power on the model. For example if a model rebuilt twice on two overlapping separate samples with a 90:10 ratio resulted in 89% accuracy and subsequent 15% accuracy on each respective 10% testing set, it was determined that this ratio resulted in overfitting and lack of generalizability once validated on the testing set. In contrast, the final selected ratio of 70:30 gave similar accuracies upon testing set validation across multiple re-sampled training and testing sets and multiple predictive model rebuilds.

### Validation and Evaluation of Performance

Model accuracy was tested by examining the total false positive rates, specificity, sensitivity on the designated testing set. Model generalizability was tested by cross validation on the testing set. Cross validation is a resampling method used to evaluate machine learning models such as logistic regression on limited data samples. Cross-validation seeks to understand the model's ability to predict new data that was not used in creation of the model. Cross-validation helps identify common problems such as overfitting and selection bias to evaluate how the predictive model might perform in practice. The model was cross validated 10 times using 10 subgroups (k-cross validation with *k* = 10) randomly drawn from the 30% test set (out-of-sample testing) to ensure generalizability. This cross-validation was done only within the testing set and included no data from the training set. Although for each partition the same training data and model is used, the 10 subgroups sampled from the test set are non-overlapping. Cross-validation was done for every predictive model rebuild on every ratio of training:testing set data and every re-sampled training set. The average results from the cross-validations was used to estimate the model's predictive performance on future datasets. The averaged set of cross-validated outcomes provides a more accurate estimate of a model's predictive capability (Grossman et al., 2010).

The predictive model was iteratively re-built until the best set of inputs and model coefficients remained to allow for a high rate of accuracy and generalizability, or ability to be applied to new populations and maintain the same result. This means that the predictive model was built on multiple different re-sampled ratio's of training: testing set samples. For each training to testing split, the training set was also re-sampled from the original sample and subsequently validated and cross-validated on its respective testing set. The final result and model outcome was the ability of the combination of independent variables (model features) to predict the dependent binary variable (CFS vs. control status).

To test the significance of model accuracy, the models were then subjected to a “Shuffle Test.” The labels on the subjects (CFS or SC) were shuffled in python using the built in sample function and passed through the model process 1,000 additional times to test if the original accuracy could be incurred by random chance. Each of the 1,000 runs trained the original model coefficients on a randomly selected stratified sample of 70% of each respective new shuffled sample (training set) and then tested the resultant model on a 30% randomly selected stratified sample (testing set) from this shuffled set to mimic the original conditions. The process was repeated on the entire shuffled sample 1,000 times. This process was repeated an additional 10,000 times if no accuracy greater than or equal to the original model accuracy could be obtained. If the Shuffle Test produced the model accuracy greater than or equal to the original model accuracy <5% of the time, it was determined the model was significant at the *p* < 0.05 level.

Logistic model coefficients must be treated with care. The logistic coefficient quantifies the rate of change in the “log odds” of the dependent variable as the input variable changes. The y-intercept term (β_0_) is the log-odds of an outcome variable when all predictors are 0. In a multivariate model, the coefficients represented by β_1_ to β_i_ show the increase in log-odds relative to each other. For example, a coefficient of β_i_ = 1 multiplies the odds of x_i_ by 10^1^ = 10, while a coefficient of 2 multiples the odds by 10^2^ = 100. These coefficients are highly dependent on other variable inputs to the model. A negative coefficient could indicate a negative relationship with the outcome variable and surrounding variables just as a positive coefficient could indicate a positive relationship, however one cannot ascertain the direction of correlation between any pair of variables in the outcome due to the nature of multivariate models and interactions between multiple variables.

### Visualization

The Wake Forest PICK ATLAS was used to select the AAL regions that contributed to each significant model and then data were imported into marsbar. These were displayed as color-coded axial slices (MRIcron).

Pearson's correlation coefficient was used to visually examine differences between CFS and sedentary control on Days 1 and 2 (Before and after exercise).

## Results

### Demographics

All subjects had a sedentary lifestyle with <40 min of active aerobic work or exercise per week. The subjects spanned a similar age and BMI range, however due to the wider range of ages in the control group, age and gender were controlled in the final model build. CFS had significantly worse symptoms (Baraniuk et al., 2013) and quality of life (Ware and Gandek, 1998; Table 1).

**Table 1:** Demographics (mean ± SD).

**Group**	**SC**	**CFS**
N	31	38
Age	43.9 ± 16.3	47.74 + 16.46
BMI	28.4 ± 4.5	26.20 + 4.52
Male	19 (61.3%)	10 (26.3%)^†^
White	23 (74.2%)	34 (89.4%)^†^
**CFS symptom severity scores**		
Fatigue	1.2 ± 1.0	3.4 + 0.8^**^
Memory and concentration	1.0 ± 1.2	2.9 + 0.9^**^
Sore throat	0.2 ± 0.6	1.0 + 1.0^*^
Sore lymph nodes	0.1 ± 0.4	1.0 + 1.1^*^
Muscle pain	0.6 ± 0.9	2.5 + 1.3^**^
Joint pain	0.8 ± 1.0	1.8 + 1.4^*^
Headaches	1.0 ± 1.3	2.0 + 1.3^*^
Sleep	1.7 ± 1.4	3.2 + 0.9^**^
Exertional exhaustion	0.5 ± 1.0	3.5 + 0.8^**^
**MOS SF-36**		
Physical functioning	88.8 ± 21.1	46.2 ± 26.3^**^
Role physical	86.8 ± 31.5	9.2 ± 25.0^**^
Bodily pain	85.9 ± 19.2	46.7 ± 26.7^**^
General health	73.8 ± 21.9	34.6 ± 23.4^**^
Vitality	64.9 ± 20.8	18.9 ± 15.7^**^
Social functioning	85.3 ± 22.1	32.6 ± 27.0^**^
Role emotional	90.2 ± 27.9	70.2 ± 44.4
Mental health	76.1 ± 16.9	67.6 ± 16.8
Chalder fatigue score	12.1 ± 4.5^**^	22.8 ± 6.4^**^

* *Scale: 0 = none, 1 = trivial, 2 = mild, 3 = moderate, 4 = severe. Mean ± SD*.

^*^p < 0.001 and

** *p < 0.000001 by 2-tailed unpaired Student's t-tests with Bonferroni corrections*;

† *p < 0.001 by Fisher's Exact Test*.

### Selection of Threshold

**Figure 1:**
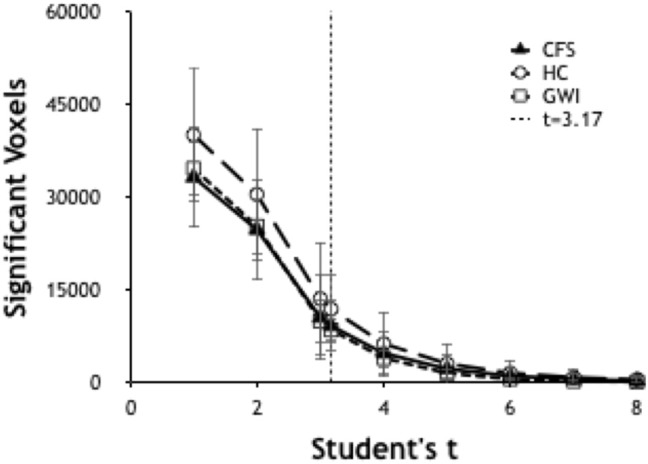
Number of significant voxels plotted vs. *T*-values for each group. The number of significant voxels decreases at higher *T*-values and more rigorous *p*-values. *T* of 3.17 indicated *p* < 0.001.

Significant voxels were identified by calculating the number of voxels per brain scan at different levels of significance. Ultimately it was determined to use a threshold of *T* ≥ 3.17 (*p* ≤ 0.001) (Figure 1) due to its ability to allow a workable number of voxels while preserving significance.

### Feature Selection

**Figure 2:**
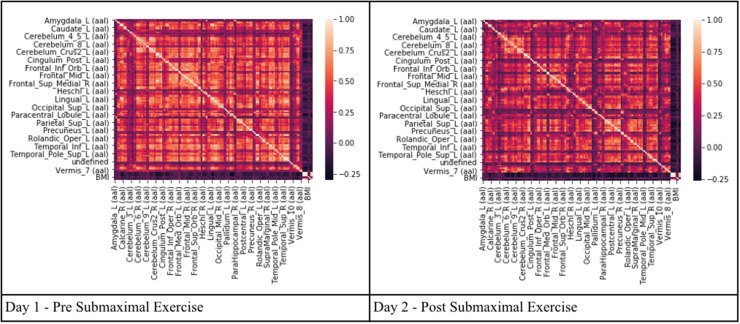
Heat maps depicting Pearson's correlation coefficients (*R*) for all AAL regions in CFS and control datasets. The diagonal white line indicates *R* = 1. The x and y axis correspond to different regions of the brain according to the AAL atlas respectively, such that the diagonal line should be a perfect correlation (One region measured against itself) and the remaining are the cross product of the rest.

Pearson's correlation coefficients were calculated between all AAL regions in the combined CFS and control dataset. All correlation coefficients were below 0.8 indicating that there was no collinearity between AAL regions or no significant dependency within model parameters on Day 1 and Day 2 for the groups (Figure 2). All regions were retained in the model because *R* was less than the cutoff point of 0.9 needed to justify removal. Although all regions were included, ultimately many of the 117 AAL regions were later removed in the recursive feature selection step and logistic regression.

### Model Results

#### Significantly Activated Regions

**Figure 3:**
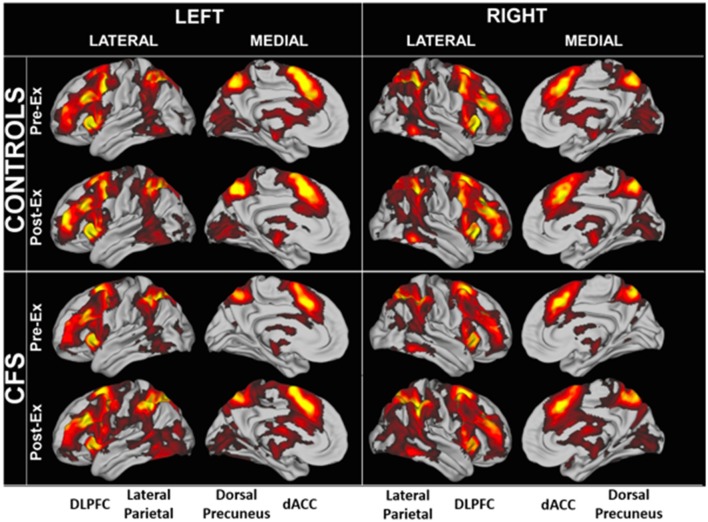
Significantly elevated BOLD activity during the 2 > 0 back condition in CFS and control groups before and after exercise.

**Figure 4:**
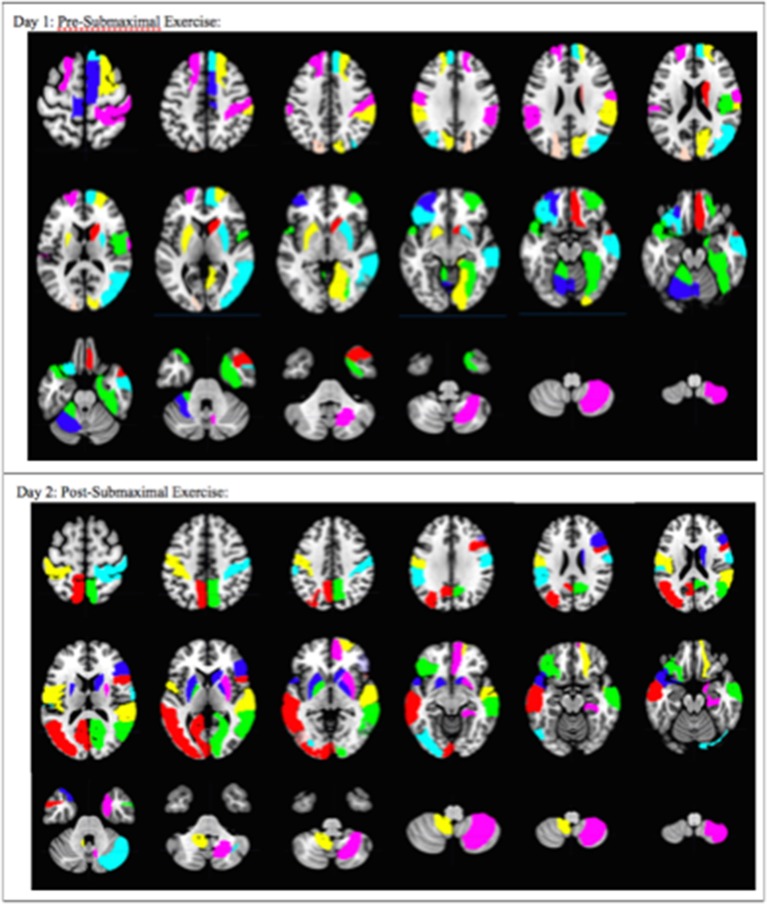
Overall pattern depicting the difference in brain activation between CFS and sedentary control groups on Day 1 and Day 2. Axial slices show the pattern of 29 AAL regions that had significantly different numbers of activated voxels (*t* > 3.17, *p* < 0.001) in the 2 > 0-black condition based on logistic regression analysis. The complete pattern reflects the overall changes in all regions. Individual AAL regions are color coded for clarity. The colors do not indicate differences in BOLD signal intensity, *t*-values, logistic regression coefficients or Pearson's correlation coefficients for any single region between the two groups.

Region of interest analysis identified areas that were significantly activated in each group (Figure 3). BOLD patterns for the 2-back > 0-back residual condition (2 > 0-back condition) were similar between CFS and controls and between Days 1 and 2 (Figure 4). Bilateral dorsolateral prefrontal cortex extending to the anterior insulae, dorsal anterior cingulate cortex, lateral parietal, and dorsal medial precuneus were activated. These match frontal parietal executive control, anterior salience, and dorsal attention networks (Laird et al., 2011; Rottschy et al., 2012). Exercise did not cause significant changes in BOLD of these regions.

#### Differentially Activated Regions Found by Predictive Model Build

Logistic regression and recursive feature elimination identified three general patterns for regions that were differentially activated between CFS and controls. Ten AAL regions were selected by the logistic regression models on both days (Table 2), suggesting these ten regions may represent persistent indicators of CFS pathologies. In addition, 19 were significant only on Day 1, and 18 only on Day 2.

**Table 2:** AAL regions and logistic regression coefficients.

**AAL ID**	**AAL abbreviation**	**Day 1**	**Day 2**
72	R Caudate_R (CAU)	0.054	0.010
73	L Putamen_L (PUT)	−0.375	0.065
74	R Putamen_R (PUT)	−0.449	0.010
63	L Supramarginal gyrus (SMG)	0.294	−0.008
58	R Postcentral gyrus (POST)	−0.379	−0.161
40	R Parahippocampus (PHIP)	0.092	0.015
15	L Inferior frontal gyrus, orbital (F3O)	0.142	0.214
86	R Middle temporal gyrus (T2)	−0.106	−0.153
83	L Temporal pole; superior temporal gyrus (T1P)	−0.128	−0.427
104	R Cerebellum 8	0.115	−0.028
3	L Superior frontal gyrus, dorsolateral (F1)	−0.117	
4	R Superior frontal gyrus, dorsolateral (F1)	0.107	
24	R Superior frontal gyrus, medial (F1M)	0.303	
10	R Middle frontal gyrus, orbital (F2O)	−0.262	
28	R Gyrus rectus (GR)	−0.081	
9	L Middle frontal gyrus, orbital (F2O)	0.193	
88	R Temporal pole; middle temporal gyrus (T2P)	0.000	
64	R Supramarginal gyrus (SMG)	0.206	
97	L Cerebellum 4 5	0.340	
112	R Vermis 6	−0.374	
99	L Cerebellum 6	−0.278	
20	R Supplementary motor area (SMA)	−0.145	
69	L Paracentral lobule (PCL)	−0.176	
18	R Rolandic operculum (RO)	0.534	
46	R Cuneus (Q)	0.566	
48	R Lingual gyrus (LING)	−0.292	
49	L Superior occipital lobe (O1)	0.290	
52	R Middle occipital lobe (O2)	−0.262	
56	R Fusiform gyrus (FUSI)	0.269	
75	L Pallidum_L (PAL)		−0.172
76	R Pallidum_R (PAL)		−0.062
43	L Calcarine fissure and surrounding cortex (V1)		−0.134
44	R Calcarine fissure and surrounding cortex (V1)		0.122
51	L Middle occipital lobe (O2)		0.203
53	L Inferior occipital lobe (O3)		−0.209
82	R Superior temporal gyrus (T1)		0.252
12	R Inferior frontal gyrus, opercular (F3OP)		0.139
14	R Inferior frontal gyrus, triangular (F3T)		−0.148
6	R Superior frontal gyrus, orbital (F1O)		−0.039
26	R Superior frontal gyrus, medial orbital (F1MO)		−0.178
67	L Precuneus (PQ)		−0.172
68	R Precuneus (PQ)		0.107
85	L Middle temporal gyrus (T2)		0.091
17	L Rolandic operculum (RO)		0.542
57	L Postcentral gyrus (POST)		0.071
92	R Cerebellum crus 1		0.086
105	L Cerebellum 9		0.095

*Ten regions were differentially activated between CFS and SC on both Days 1 and 2, with 17 regions only on Day 1 and 16 other regions only after exercise*.

These 10 AAL regions were the right caudate, left and right putamen, left supramarginal gyrus (SMG), right postcentral gyrus (POST), right parahippocampus (PHIP), left inferior frontal gyrus orbital (F3O), right middle temporal gyrus (T2), left temporal pole; superior temporal gyrus (T1P), and the right cerebellum 8.

The 19 regions significant on Day 1 only were the left superior frontal gyrus; dorsolateral (F1), right superior frontal gyrus; dorsolateral (F1), right superior frontal gyrus; medial (F1M), right middle frontal gyrus; orbital (F2O), right gyrus rectus (GR), left middle frontal gyrus; orbital (F2O), right temporal pole; middle temporal gyrus (T2P), right supramarginal gyrus (SMG), left cerebellum 4 5, right vermis 6, left cerebellum 6, right supplementary motor area (SMA), left paracentral lobule (PCL), right rolandic operculum (RO), right cuneus (Q), right lingual gyrus (LING), left superior occipital lobe (O1), right middle occipital lobe (O2), right fusiform gyrus (FUSI).

The 18 regions significant on Day 2 only were the left and right pallidum (PAL), left and right calcarine fissure and surrounding cortex (V1), left middle occipital lobe (O2), left inferior occipital lobe (O3), right superior temporal gyrus (T1), right inferior frontal gyrus opercular (F3OP), right inferior gyrus triangular (F3T), right superior frontal gyrus orbital (F10), right superior frontal gyrus medial orbital (F1MO), left and right precuneus (PQ), left middle temporal gyrus (T2), left rolandic operculum (RO), left postcentral gyrus (POST), right cerebellum crus 1, and left cerebellum 9.

**Figure 5:**
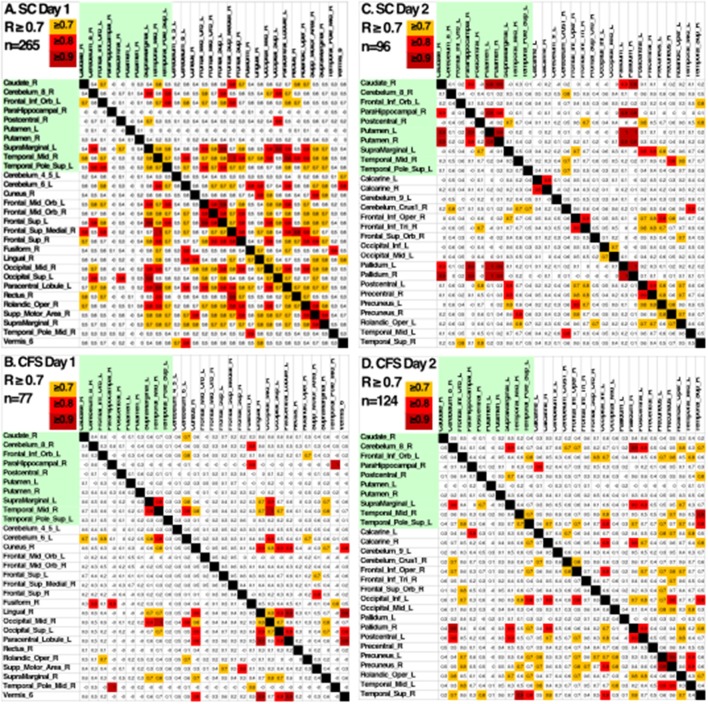
Pearson's correlation coefficients between the BOLD activities in AAL regions that were differentially activated in SC and CFS on Days 1 and 2. AAL regions were listed in alphabetical order on the x- and y-axes for the 10 regions that were activated on Days 1 and 2 (yellow), on only on Day 1 or Day 2. Correlations were color coded in orange (*R* > 0.7), red (*R* > 0.8) and dark red (*R* > 0.9). As predicted by the logistic regression, CFS and SC had different patterns of correlations for the 10 shared regions (green) on Days 1 and 2, and between CFS and SC for the regions that were significantly different on Day 1 and Day 2.

Another indication of significant differences was shown by looking at the patterns of Pearson's correlation coefficients between individual AAL regions between groups and days (Figure 5). SC on Day 1 had the highest number of correlations with *R* ≥ 0.7 suggesting that control subjects were focused on the task on Day 1. SC subjects had fewer correlations with *R* > 0.7 on Day 2 suggesting that they exhibited learning, automaticity, and required a lower level of focus to complete the n-back task. CFS had fewer correlations on the pre-exercise MRI scan, different patterns of correlations from SC on both days, and poor similarity between Days 1 and 2. The different patterns of correlations between AAL regions supported the logistic regression analysis and demonstrated differences in connectivity between brain regions for CFS and SC before and after exercise. These outcomes predict that more advanced measures of functional connectivity (Rubinov and Sporns, 2010) will differ between CFS and SC before and after exercise and when depicting changes related to post-exertional malaise.

The composite multivariate pattern of activation differentiated CFS from Control with 80.9% accuracy on Day 1 and 76.1% accuracy on Day 2. Cross validation performed better than random on both days with a 65% accuracy on Day 1 and 57.5% accuracy on Day 2 (Table 3). Both the Day 1 and Day 2 models were able to correctly predict CFS from a SC greater than random chance (>0.5) due to this high predictive performances, however the Day 1 predictive model showed greater predictive power than the Day 2 model upon cross-validation. More samples in a future study would assist in validating this predictive performance.

**Table 3:** Model results for day 1 (pre-submaximal exercise) and day 2 (post submaximal exercise).

	**Pre-exercise (day 1)**	**Pre-exercise (day 2)**
Accuracy	80.9%	76.1%
10x cross validation frequency	65%	57.5%
Sensitivity	87.5%	76.9%
Specificity	76.9%	75%
PPV	70%	83.3%
NPV	90.9%	66.7%
Significance as determined from Shuffle Test	*p* < 0.01	*p* < 0.05
Shuffle test average	44%	46%
Shuffle test mode	37.5%	43.5%

*The accuracy and 10x cross validation frequency represent the corresponding model accuracy and accuracy after being ran through 10 smaller sub samplings of the initial testing set*.

The Shuffle Test reproduced an accuracy of 65% on 0 of the 1,000 shuffled test runs for Day 1. The Day 1 Shuffle Test had an average of 44% accuracy and mode of 37.5% accuracy for the 1,000 test runs. To ensure the statistical rigor of this method, the Shuffle Test was repeated for an additional 10,000 permutations on Day 1. A maximum accuracy of 69% was obtained and results for 65% accuracy or greater were found 11 times of 10,000 runs. Thus, it was determined the Day 1 model was significant at a *p* < 0.01 level. The Shuffle Test for Day 2 reproduced an accuracy of 57% or higher on 40 of 1,000 test runs. The Day 2 Shuffle Test had an average of 46% accuracy and mode of 43.5% accuracy for the 1,000 test runs. As both tests reproduced the model accuracy on <5% of 1,000 shuffled runs, it was determined that each model was significant at *p* < 0.05.

## Discussion

This machine-learning approach was able to uncover a pattern of activated neurological regions that differentiated CFS from control subjects. The results of these two models indicate that machine learning algorithms combined with the voxel counts for activated regions grouped into the AAL Atlas was able to differentiate CFS from sedentary controls with good accuracy. The outcome indicates that analysis of fMRI data by machine learning algorithm(s) may lead to their use as part of a diagnostic tool that relies on cognitive aspects of CFS and their response to the physiological stressor of exercise. This may provide objective support for the concept of post-exertional malaise that is a central tenet of current subjectively defined CFS diagnostic criteria (Fukuda et al., 1994; Carruthers et al., 2003).

Ten AAL regions were significantly different according to the predictive model between SC and CFS before and after exercise and may represent persistent indicators of CFS pathologies. Left and right putamen and right caudate of the basal ganglia may be part of the Affective Network that has been identified by meta-analysis of studies in anxiety (Xu et al., 2019). The primary sensory region (right S1) has been associated with heightened sensory awareness in panic disorder (Kim and Yoon, 2018). AAL regions of the ventromedial prefrontal cortex, temporal lobe, and parahippocampus overlapped with nodes from the default mode network (DMN) (Fox et al., 2015). They may be more related with subsets of the DMN related to rest and retrieval than forward thinking (Bellana et al., 2017). The left cerebellar hemisphere region eight has motor functions but is adjacent to regions having cognitive effects (Schmahmann, 2019).

Nineteen regions distinguished CFS from control only on Day 1 before exercise. They fit into several general patterns. Ventromedial and dorsomedial prefrontal cortex, parahippocampus and temporal pole regions are part of the default mode network (Mazziotta et al., 1995; Fox et al., 2015; Bellana et al., 2017). Cerebellar regions mediated working memory and emotional processing, and may have interacted with supplementary motor areas in pain and interoceptive dysfunction (Schmahmann, 2019). Occipital regions implicated visual functions. Bilateral supramarginal gyri suggested a role in the systemic hyperalgesia found in CFS (Lanz et al., 2011).

These 29 regions were differentially activated in CFS and controls before exercise. They included six ventromedial and dorsomedial frontal cortex regions of the anterior division of the DMN, and the right cuneus, right middle temporal gyrus (T2P), and right supramarginal gyrus from the posterior DMN (Laird et al., 2009; Fox et al., 2015). Heightened sensory awareness was implicated by activation of five regions of the visual network, right Rolandic operculum, supplementary motor areas (SMA), and cerebellum. The Rolandic operculum is the “little lid” of the parietal lobe that folds over the posterior insula. The bilateral Rolandic operculum integrates exteroceptive and interoceptive signals that are necessary for bodily self-consciousness and interoceptive awareness (Wager et al., 2013; Blefari et al., 2017) and is activated for maintenance of vigilant attention during simple tasks such as the stimulus-response 0-back task and discrimination tasks that require continuous decisions about alternative responses (e.g., go vs. no-go tasks) (Langner and Eickhoff, 2013).

The right supplementary motor area (SMA) and left paracentral lobule are functionally connected to cerebellar regions during pain processing (Coombes and Misra, 2016). Left cerebellar hemispheres 4, 5, and 6 have been implicated in working memory and generalized aversive processing, while right vermis six functions in emotional processing (Schmahmann, 2019). Visual regions may be differentially activated for attention (Vossel et al., 2014) or visual memory (Baldassano et al., 2016) during the n-back task.

After exercise, 18 other regions were activated. They included bilateral pallidum, precuneus, and superior frontal gyri, and visual cortex. These 28 regions were differentially activated on Day 2. Left and right pallidum joined other basal ganglia regions of the affective network (Xu et al., 2019). Bilateral precuneus, anterior insula, and sensorimotor cortex (Vossel et al., 2014), ventromedial frontal cortex and temporal regions suggest activation of the rostromedial frontal—lateral temporal subnetwork of the DMN (Bellana et al., 2017). Left and right precuneus may indicate DMN activation, or recruitment for cognitive compensation during the challenging 2-back task. Even though the dorsal precuneus is a node in the DMN, it can be recruited into task systems during n-back testing (Rzucidlo et al., 2013). This is in contrast to the ventral precuneus that is only associated with DMN functions. Sensory activation was suggested by activation of bilateral postcentral gyri (S1) as found in panic disorder (Kim and Yoon, 2018). Occipital lobe visual network regions were particularly noteworthy and may indicate heightened vigilance, visual and memory analysis, or general sensory hypersensitivity. The ventral attention network was suggested by activation of the right ventrolateral prefrontal cortex (Fox et al., 2015). Attention and vigilance were implied from the activation of visual regions that can interact with dorsal attention network nodes in the intraparietal sulcus, and the right temporal parietal junction of the ventral attention network (Vossel et al., 2014). The left Rolandic operculum had the highest coefficient of any region, and was notable for its association with bodily self-consciousness, interoceptive and pain networks (Blefari et al., 2017).

The specific AAL regions that were differentially activated and selected by the logistic regression model were different between CFS and control on Days 1 and 2, but many of the regions were closely related because they belonged to the same functionally defined brain networks. Many belonged to the default mode network (DMN). Differential activation was found in the ventromedial and dorsomedial prefrontal cortex, hippocampus, lateral and temporal poles of the DMN, but with no significant differences for the medial posterior DMN nodes in the retrosplenial and posterior cingulate cortex regions (Laird et al., 2011; Fox et al., 2015). Affective network regions included basal ganglia, dorsal precuneus, sensorimotor regions, dACC and anterior insulae (Kim and Yoon, 2018). However, the amygdala was not differentially activated in CFS vs. control. The logistic regression included cerebellar and supplementary motor regions involved in working memory suggesting they were recruited as cognitive compensation or because of their interactions with sensorimotor regions during pain processing (Schmahmann, 2019). Cognitive compensation was suggested by the inclusion of the dorsal precuneus in the logistic regression on Day 2. Multimodal sensory intergration was suggested by visual and sensorimotor nodes, and on Day 2 by the addition of the ventral attention network. The Rolandic operculum, affective, cognitive, sensory, and attention network changes on Day 2 after exercise provocations may point to regions involved in post-exertional malaise in CFS. Involvement of these networks in the logistic regression was consistent with attention, memory and other cognitive dysfunction, chronic pain, systemic hyperalgesia and allodynia, negative emotion, and labile arousal that are part of the clinical presentation of CFS.

A limitation was the small sample size that created relatively small training and validation sets. The results of this pilot study can now be used to power larger studies to test the hypotheses proposed above. The nature of logistic regression means that individual regions of activation or deactivation of pathological significance for CFS cannot be determined from the model results alone. The coefficients assigned to input features are the “log odds” for the statistical models and not actual representations of increased or decreased BOLD activities. Because the variables depend on one another, it is the collective grouping of all AAL regions from the regression that ultimately show the difference between CFS and control. It is the entire pattern that transforms the fMRI data into a potential diagnostic biomarker. This methodology may be generalizable to allow sharing of fMRI data and creation of a diagnostic tool.

## Conclusion

The logistic regression model performed on fMRI data significantly differentiated CFS from control with model accuracy of 80.9% on Day 1 before exercise and 76.1% on Day 2 during the period of post-exertional malaise. Before exercise, CFS and control groups were different because of differential activation in default mode network nodes, and sensory perception networks involving visual, somatic, supplementary motor areas and cerebellar regions. These differences suggested dysfunction of attention and potential distraction by sensory processing in pain and interoception. Differential activation after exercise may indicate objective alterations related to post-exertional malaise involving frontal and lateral temporal nodes of the default mode network, sensory hypervigilance and attention using the left Rolandic operculum, visual network and the ventral attention network, and basal ganglia in the Affective Network.

## Data Availability Statement

The final compiled data that was generalized and analyzed for this study can be found within article tables. Individual patient records are not published, however could be de-identified and made available upon request.

## Ethics Statement

The studies involving human participants were reviewed and approved by Georgetown University Institutional Review Board (IRB 2009-229, 2013-0943, 2015-0579) and U.S. Army Medical Research and Material Command (USAMRC) Human Research Protection Office (HRPO A-155547.0, A-18749). The patients/participants provided their written informed consent to participate in this study.

## Author Contributions

The predictive model experiment design, predictive model build, data analysis, visualization, and final article write up were performed by DP. SW performed data pre-processing, visualization, and review of article write up. JB performed initial data collection, visualization, article review, and project oversight.

### Conflict of Interest

The authors declare that the research was conducted in the absence of any commercial or financial relationships that could be construed as a potential conflict of interest.

## Footnotes

^1^http://www.pstnet.com/eprime.cfm

^2^http://www.alivelearn.net/xjview/

^3^https://figshare.com/articles/_Abbreviations_and_MNI_coordinates_of_AAL_/184981

^4^http://scikit-learn.org/stable/modules/feature_selection.html#rfe
