# Supplementary material for: A Machine Learning Approach to the Differentiation of Functional Magnetic Resonance Imaging Data of Chronic Fatigue Syndrome (CFS) From a Sedentary Control
Source: Front Comput Neurosci. 2020 Jan 29;14:2. doi: 10.3389/fncom.2020.00002 (PMC7000378; doi:10.3389/fncom.2020.00002)

Supplementary Material

SOM. Table S1. AAL Atlas centers of mass (MNI coordinates) and voxels per region. https://figshare.com/articles/_Abbreviations_and_MNI_coordinates_of_AAL_/184981

Index	L R	Abbreviation	X	Y	Z	Voxels
1	L	Precentral_L	−39	−6	51	3526
2	R	Precentral_R	41	−8	52	3381
3	L	Frontal_Sup_L	−18	35	42	3599
4	R	Frontal_Sup_R	22	31	44	4056
5	L	Frontal_Sup_Orb_L	−17	47	−13	963
6	R	Frontal_Sup_Orb_R	18	48	−14	997
7	L	Frontal_Mid_L	−33	33	35	4863
8	R	Frontal_Mid_R	38	33	34	5104
9	L	Frontal_Mid_Orb_L	−31	50	−10	888
10	R	Frontal_Mid_Orb_R	33	53	−11	1015
11	L	Frontal_Inf_Oper_L	−48	13	19	1038
12	R	Frontal_Inf_Oper_R	50	15	21	1399
13	L	Frontal_Inf_Tri_L	−46	30	14	2529
14	R	Frontal_Inf_Tri_R	50	30	14	2151
15	L	Frontal_Inf_Orb_L	−36	31	−12	1690
16	R	Frontal_Inf_Orb_R	41	32	−12	1707
17	L	Rolandic_Oper_L	−47	−8	14	990
18	R	Rolandic_Oper_R	53	−6	15	1331
19	L	Supp_Motor_Area_L	−5	5	61	2147
20	R	Supp_Motor_Area_R	9	0	62	2371
21	L	Olfactory_L	−8	15	−11	280
22	R	Olfactory_R	10	16	−11	289

23	L	Frontal_Sup_Medial_L	−5	49	31	2992
24	R	Frontal_Sup_Medial_R	9	51	30	2134
25	L	Frontal_Med_Orb_L	−5	54	−7	719
26	R	Frontal_Med_Orb_R	8	52	−7	856
27	L	Rectus_L	−5	37	−18	852
28	R	Rectus_R	8	36	−18	745
29	L	Insula_L	−35	7	3	1858
30	R	Insula_R	39	6	2	1770
31	L	Cingulum_Ant_L	−4	35	14	1400
32	R	Cingulum_Ant_R	8	37	16	1313
33	L	Cingulum_Mid_L	−5	−15	42	1941
34	R	Cingulum_Mid_R	8	−9	40	2203
35	L	Cingulum_Post_L	−5	−43	25	463
36	R	Cingulum_Post_R	7	−42	22	335
37	L	Hippocampus_L	−25	−21	−10	932
38	R	Hippocampus_R	29	−20	−10	946
39	L	ParaHippocampal_L	−21	−16	−21	978
40	R	ParaHippocampal_R	25	−15	−20	1132
41	L	Amygdala_L	−23	−1	−17	220
42	R	Amygdala_R	27	1	−18	248
43	L	Calcarine_L	−7	−79	6	2258
44	R	Calcarine_R	16	−73	9	1861
45	L	Cuneus_L	−6	−80	27	1526
46	R	Cuneus_R	14	−79	28	1424
47	L	Lingual_L	−15	−68	−5	2095
48	R	Lingual_R	16	−67	−4	2300

49	L	Occipital_Sup_L	−17	−84	28	1366
50	R	Occipital_Sup_R	24	−81	31	1413
51	L	Occipital_Mid_L	−32	−81	16	3270
52	R	Occipital_Mid_R	37	−80	19	2098
53	L	Occipital_Inf_L	−36	−78	−8	941
54	R	Occipital_Inf_R	38	−82	−8	989
55	L	Fusiform_L	−31	−40	−20	2310
56	R	Fusiform_R	34	−39	−20	2518
57	L	Postcentral_L	−42	−23	49	3892
58	R	Postcentral_R	41	−25	53	3823
59	L	Parietal_Sup_L	−23	−60	59	2065
60	R	Parietal_Sup_R	26	−59	62	2222
61	L	Parietal_Inf_L	−43	−46	47	2447
62	R	Parietal_Inf_R	46	−46	50	1345
63	L	SupraMarginal_L	−56	−34	30	1256
64	R	SupraMarginal_R	58	−32	34	1974
65	L	Angular_L	−44	−61	36	1173
66	R	Angular_R	46	−60	39	1752
67	L	Precuneus_L	−7	−56	48	3528
68	R	Precuneus_R	10	−56	44	3265
69	L	Paracentral_Lobule_L	−8	−25	70	1349
70	R	Paracentral_Lobule_R	7	−32	68	836
71	L	Caudate_L	−11	11	9	962
72	R	Caudate_R	15	12	9	994
73	L	Putamen_L	−24	4	2	1009
74	R	Putamen_R	28	5	2	1064

75	L	Pallidum_L	−18	0	0	293
76	R	Pallidum_R	21	0	0	280
77	L	Thalamus_L	−11	−18	8	1100
78	R	Thalamus_R	13	−18	8	1057
79	L	Heschl_L	−42	−19	10	225
80	R	Heschl_R	46	−17	10	249
81	L	Temporal_Sup_L	−53	−21	7	2296
82	R	Temporal_Sup_R	58	−22	7	3141
83	L	Temporal_Pole_Sup_L	−40	15	−20	1285
84	R	Temporal_Pole_Sup_R	48	15	−17	1338
85	L	Temporal_Mid_L	−56	−34	−2	4942
86	R	Temporal_Mid_R	57	−37	−1	4409
87	L	Temporal_Pole_Mid_L	−36	15	−34	755
88	R	Temporal_Pole_Mid_R	44	15	−32	1187
89	L	Temporal_Inf_L	−50	−28	−23	3200
90	R	Temporal_Inf_R	54	−31	−22	3557
91	L	Cerebellum_Crus1_L	−35	−67	−29	2603
92	R	Cerebellum_Crus1_R	38	−67	−30	2648
93	L	Cerebellum_Crus2_L	−28	−73	−38	1894
94	R	Cerebellum_Crus2_R	33	−69	−40	2117
95	L	Cerebellum_3_L	−8	−37	−19	136
96	R	Cerebellum_3_R	13	−34	−19	207
97	L	Cerebellum_4_5_L	−14	−43	−17	1125
98	R	Cerebellum_4_5_R	18	−43	−18	861
99	L	Cerebellum_6_L	−22	−59	−22	1694
100	R	Cerebellum_6_R	26	−58	−24	1795

101	L	Cerebellum_7b_L	−31	−60	−45	585
102	R	Cerebellum_7b_R	34	−63	−48	534
103	L	Cerebellum_8_L	−25	−55	−48	1887
104	R	Cerebellum_8_R	26	−56	−49	2308
105	L	Cerebellum_9_L	−10	−49	−46	869
106	R	Cerebellum_9_R	10	−49	−46	809
107	L	Cerebellum_10_L	−22	−34	−42	144
108	R	Cerebellum_10_R	27	−34	−41	159
109	midline	Vermis_1_2	2	−39	−20	53
110	midline	Vermis_3	2	−40	−11	228
111	midline	Vermis_4_5	2	−52	−6	665
112	midline	Vermis_6	2	−67	−15	371
113	midline	Vermis_7	2	−72	−25	194
114	midline	Vermis_8	2	−64	−34	243
115	midline	Vermis_9	2	−55	−35	174
116	midline	Vermis_10	1	−46	−32	112

Figure S1. AAL atlas depicting all regions on sedentary control brain. Figure generated in Nilearn python package.

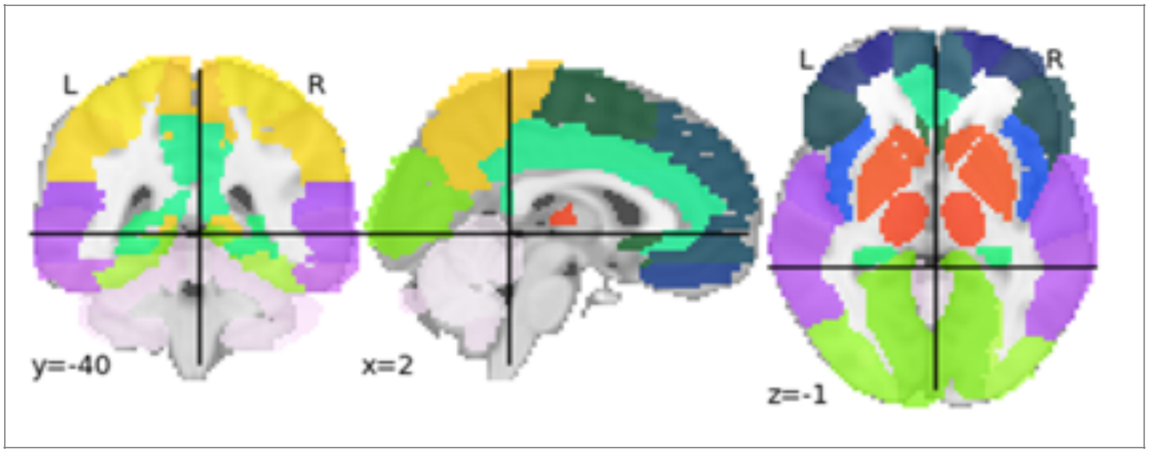

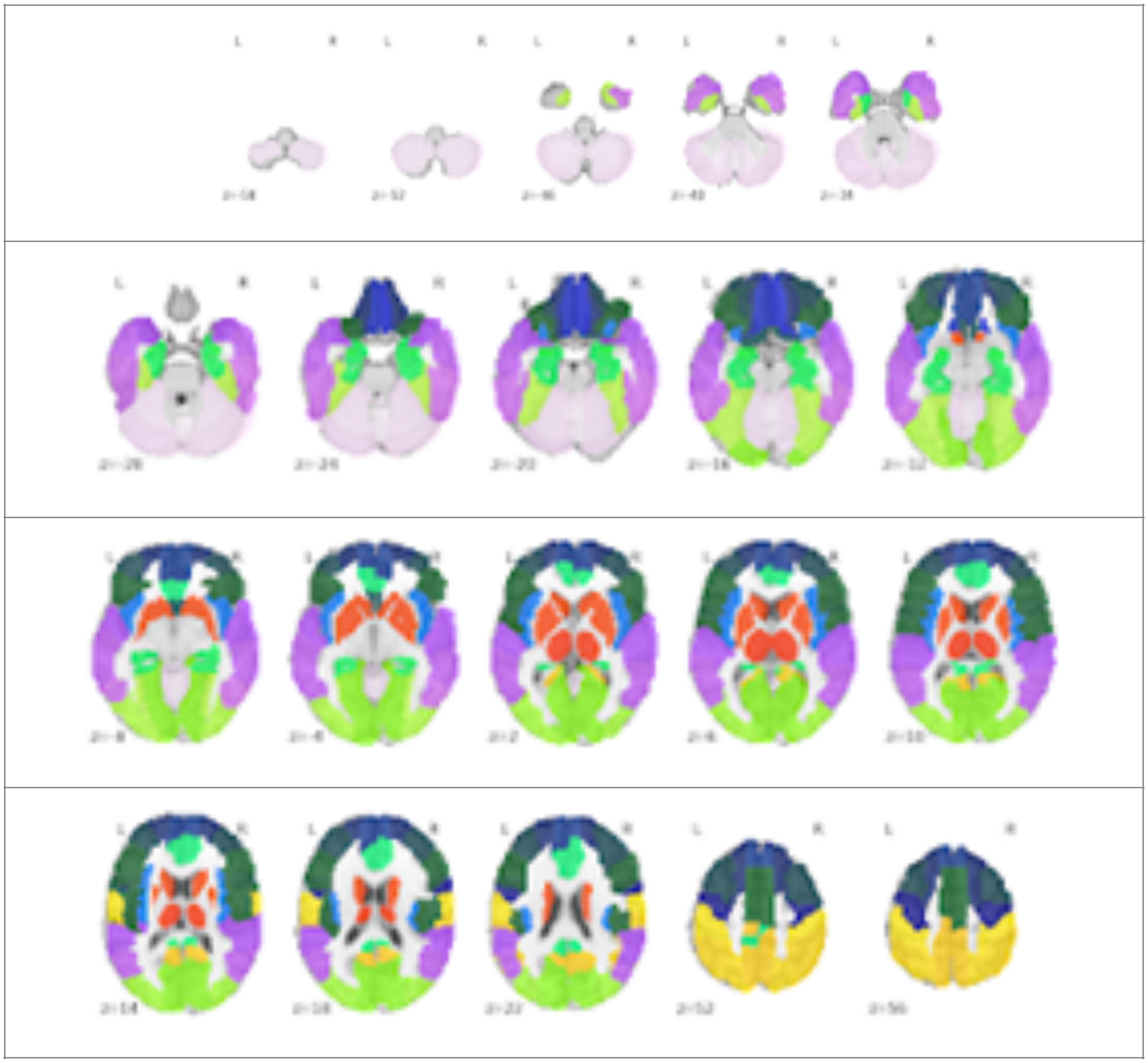

Supplement: Supplementary file 1 [file Data_Sheet_1.PDF]
